# Supplementary material for: Amplification of downstream flood stage due to damming of fine-grained rivers
Source: Nat Commun. 2022 Jun 1;13:3054. doi: 10.1038/s41467-022-30730-9 (PMC9160006; doi:10.1038/s41467-022-30730-9)
Supplement: Supplementary file 1 — Supplementary Information [file 41467_2022_30730_MOESM1_ESM.pdf]

Supplementary Information for

Amplification of downstream flood stage due to damming of fine-grained rivers

Authors: Hongbo Ma[1*], Jeffrey A. Nittrouer[2*], Xudong Fu[3*], Gary Parker[4,5], Yuanfeng Zhang[6], Yuanjian Wang[6], Yanjun Wang[3], Michael P. Lamb[7], Julia Cisneros[4], Jim Best[4,5,8,9], Daniel R. Parsons[10], Baosheng Wu[3]

Affiliations:
[1]Department of Civil and Environmental Engineering, University of California at Irvine, Irvine, California 92697, USA.
[2]Department of Geosciences, Texas Tech University, Lubbock, TX 79409.
[3]State Key Laboratory of Hydroscience and Engineering, Tsinghua University, Beijing 100084, China
[4]Department of Geology, University of Illinois at Urbana-Champaign, Illinois 61801, USA
[5]Department of Civil and Environmental Engineering, Ven Te Chow Hydrosystems Laboratory, University of Illinois at Urbana-Champaign, Illinois 61801, USA
[6]Yellow River Institute of Hydraulic Research, Zhengzhou, Henan 450000, China
[7]Division of Geological and Planetary Sciences, California Institute of Technology, Pasadena, California 91125, USA
[8]Department of Geography and Geographic Information Science, University of Illinois at Urbana-Champaign, Illinois 61801, USA
[9]Department of Mechanical Science and Engineering, University of Illinois at Urbana-Champaign, Illinois 61801, USA
[10]Energy and Environment Institute, University of Hull, Hull HU6 7RX, UK.

*Correspondence to: H.M., bigmatton@gmail.com; J.A.N., jeffrey.nittrouer@ttu.edu; X.F., xdfu@tsinghua.edu.cn.

This PDF file includes:

Figs S1 to S9
Tables S1 to S5

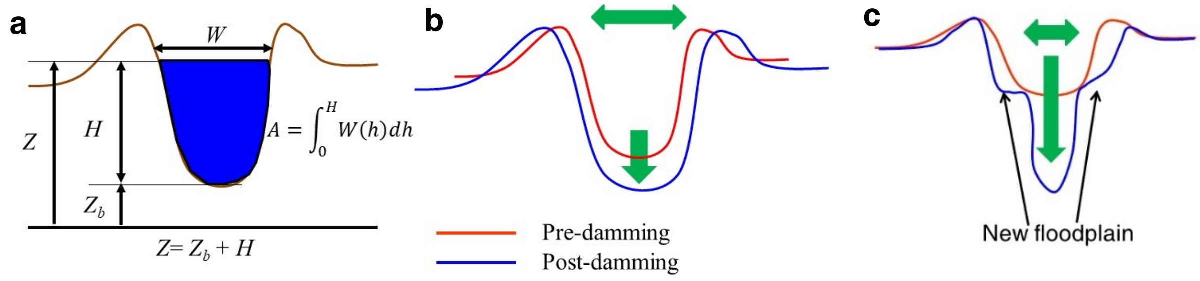

Fig. S1.

Sketch of basic parameters of channel morphology and adjustments following dam construction. **A** Basic macroscopic parameters of flow hydraulics and channel morphology; **B** Simultaneous channel deepening and widening after dam construction; **C** Relatively rapid channel deepening compared to channel widening. Note that the old channel bed is transformed into a new floodplain.

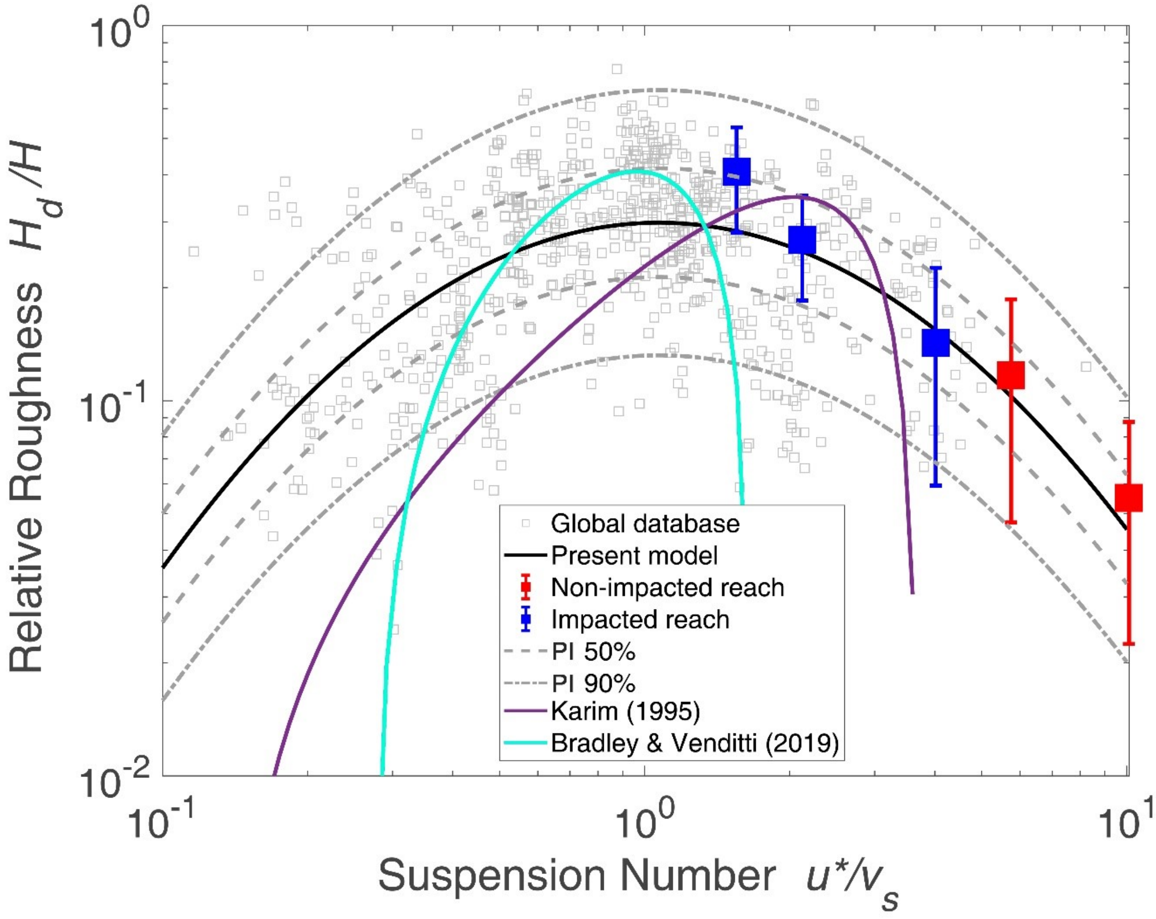

Fig. S2.

Comparison among proposed relation, compiled database and two other relations, specifying relative bedform (roughness) height versus suspension number. The proposed relation is the best-fit curve to the compiled database, with the Karim (1995)[29] and Bradley and Venditti (2019)[27] relations also plotted. The proposed relation, based on a parabolic equation in logarithmic space, i.e. $\log_{10}Y = -0.87(\log_{10}X-0.03)^2-0.53$, shows a good fit and covers a wide range of laboratory and field data. Field observations of bedform height in the dam impacted (blue squares) and non-impacted (red squares) reaches of the lower Yellow River (LYR) are plotted to test relations; the squares represent the mean value of relative bedform height and error bars represent one standard deviation. It is worth noting that despite the differences, all the relations show that post-damming bedforms in the lower Yellow River will have larger heights than the pre-damming bedforms. This plot also indicates that the critical suspension values corresponding to the maximum in dune-size are very close to each other (0.9-2.0). The hump-shaped relation indicates that if the bankfull suspension number is greater than the suspension number corresponding with the dune-size maximum (~1.1 via the present model), bed coarsening induced by damming on the river will lead to larger dunes during floods. Dashed and dash-dotted lines represent the 50% and 90% prediction intervals (PI) of the present model, respectively.

a

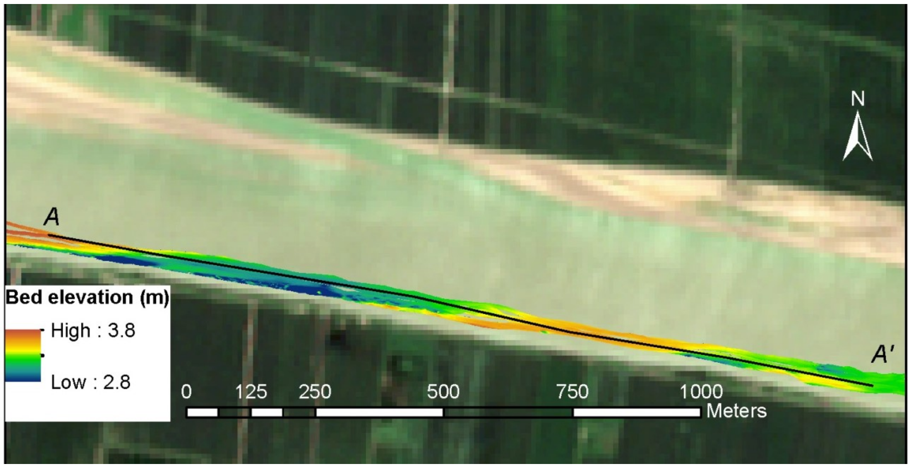
N
A
A′
Bed elevation (m)
High : 3.8
Low : 2.8
0 125 250 500 750 1000
Meters

b

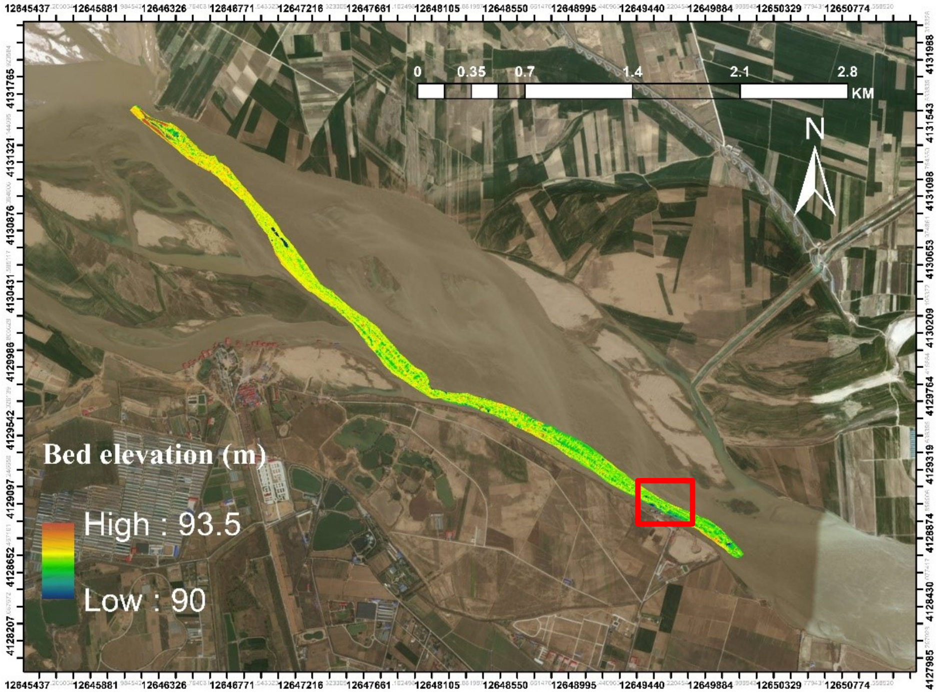
0 0.35 0.7 1.4 2.1 2.8
KM
N
Bed elevation (m)
High : 93.5
Low : 90

c

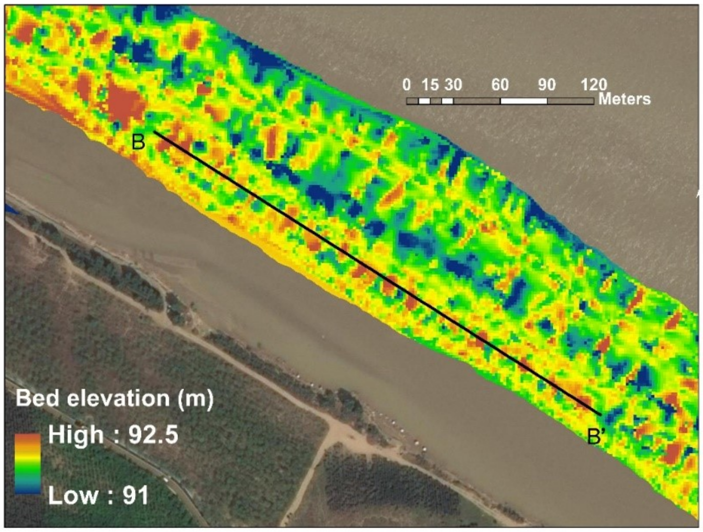
0 15 30 60 90 120
Meters
B
B'
Bed elevation (m)
High : 92.5
Low : 91

d

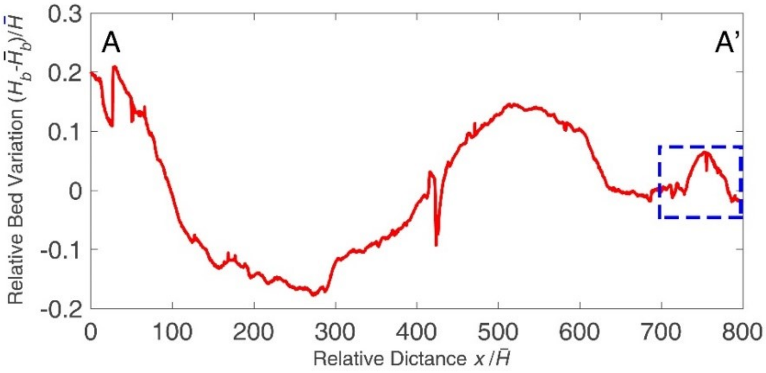
A
A'
Relative Bed Variation $(H_b-\bar{H}_b)/\bar{H}$
0.3
0.2
0.1
0
-0.1
-0.2
0 100 200 300 400 500 600 700 800
Relative Dictance $x/\bar{H}$

e

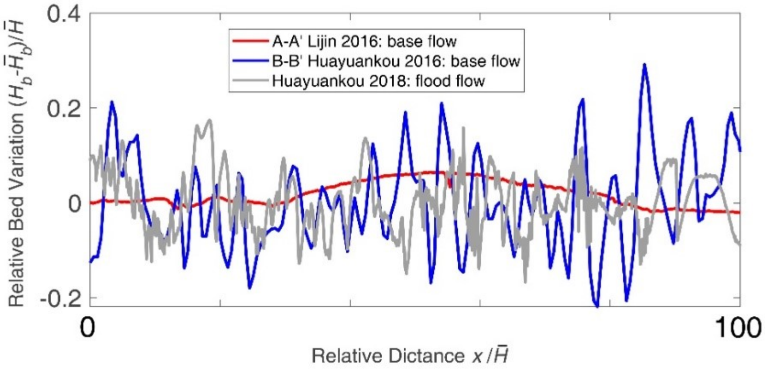
A-A' Lijin 2016: base flow
B-B' Huayuankou 2016: base flow
Huayuankou 2018: flood flow
Relative Bed Variation $(H_b-\bar{H}_b)/\bar{H}$
0.4
0.2
0
-0.2
0
100
Relative Dictance $x/\bar{H}$

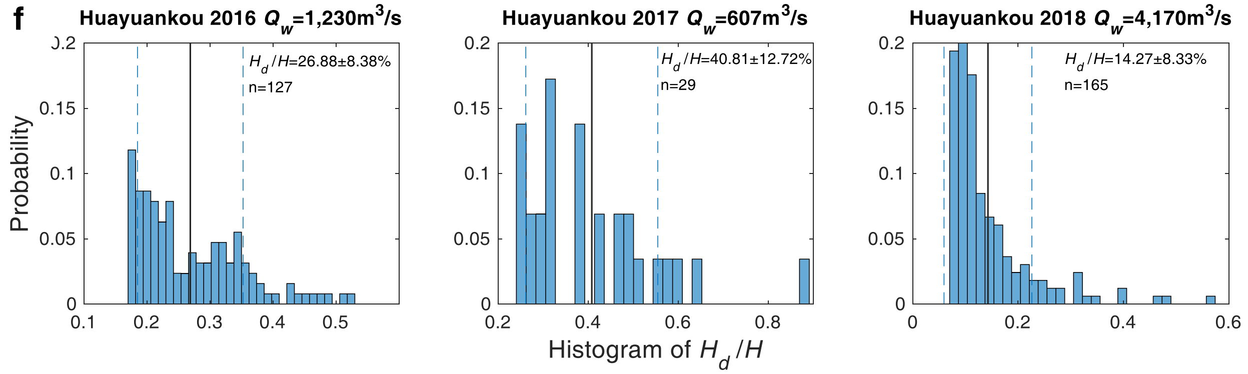

Fig. S3.

Bed topographic survey data at Lijing (non-impacted) and Huayuankou (impacted), Yellow River, China. **a** Multibeam bathymetric survey map at Lijin (base flow, 2016) shows bedforms possessing low relief and long wavelength; **b** Multibeam bathymetric survey map at Huayuankou (base flow, 2016), which is 4.5 km long and 100 m wide, shows a dune field that is distinctly different from that at Lijin; **c** Detailed MBES map of the dune field at Huayuankou; **d** Longitudinal bed profile *A-A'* (from plot **A**) at Lijin; **e** Three longitudinal bedform profiles at two studied reaches derived from MBES maps. Blue line *B-B'* is from plot **c** and red line is from the blue rectangular part of line *A-A'* in plot **d.** Gray line is the longitudinal bed profile from the Huayuankou reach under flood flow conditions. The non-impacted downstream reach (Lijin) maintains low-relief bedforms, while the channel bed of the dam-impacted reach (Huayuankou) possesses typical, high-relief dunes under base and flood flow conditions. The presence of low-relief bedforms is the primary reason for low flow resistance; **f** Statistics of dune heights at Huayuankou under different flow conditions. The statistics of bedform geometry for all bed topography surveys are summarized in Table S3.

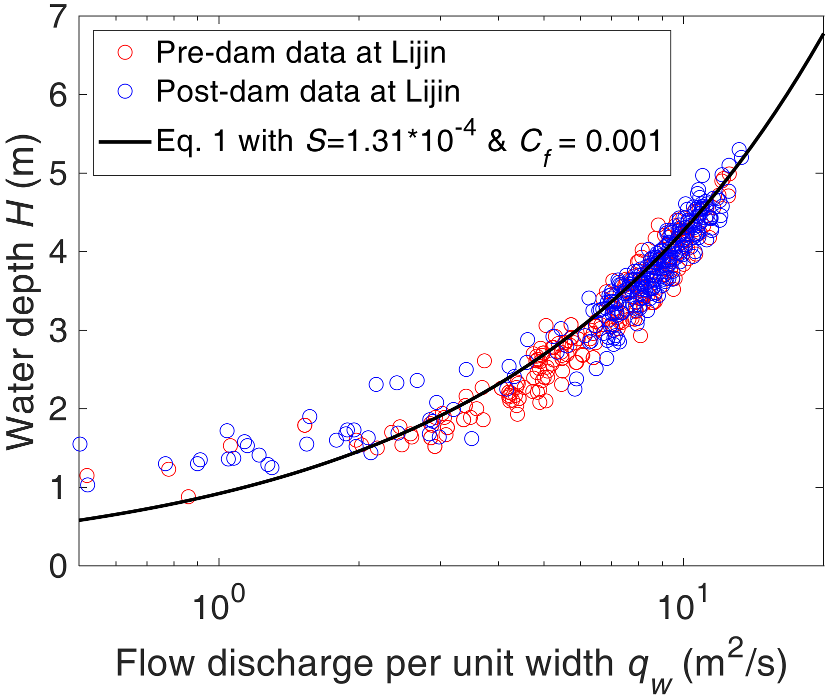

Fig. S4.

Comparison of H-q_w relations at the non-impacted reach of the Yellow River (Lijin) before and after dam construction. No change is found in the H-q_w relations between pre- and post-damming conditions. Equation 1, with channel slope S=1.31*10^{-4} and resistance coefficient C_f=0.001 (both obtained from the equilibrium data), can very well predict the data under both pre- and post-damming conditions.

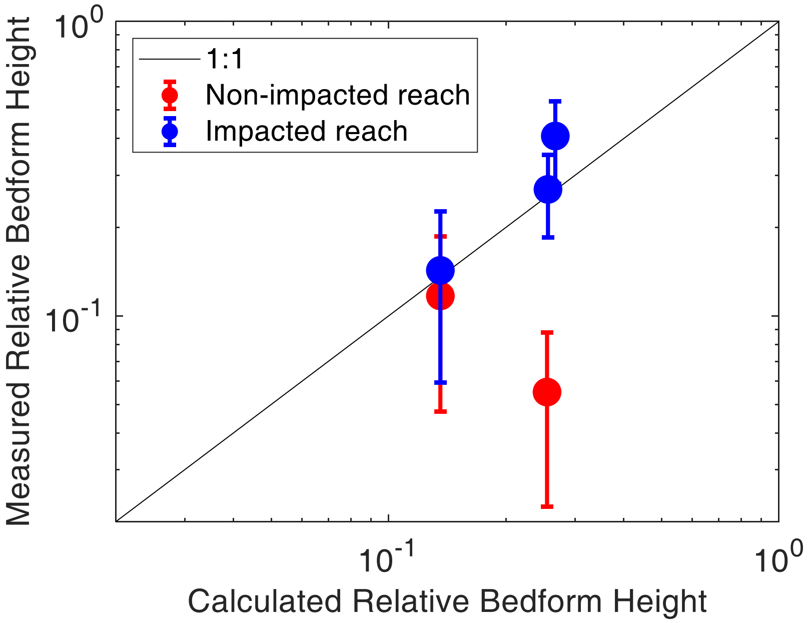

Fig. S5.

Comparison of bedform heights between measured and calculated values by Bradley and Venditti (2017)[39]. Within the five observed bedform data, one non-impacted bedform dataset (red) is significantly overestimated and one impacted bedform dataset (blue) is underestimated by Bradley & Venditti (2017)[39].

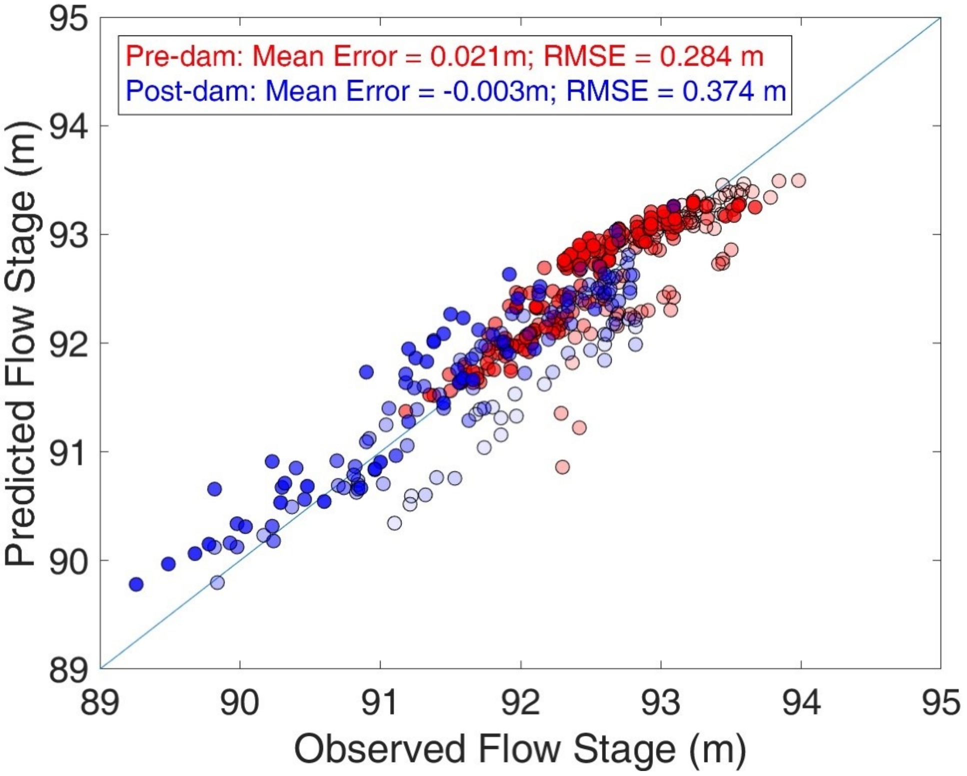

Fig. S6.

Comparison of flow stages between measured and predicted values based on cross-sectional data from each year and hydraulic model (Text S6 in Methods). The periods of the discharge-stage data are from 1981 to 1990 for pre-dam and from 2006 to 2015 for post-dam. The cross-sectional shape observed before the flood season of each year was used to predict each *Q-Z* data in that year. The lighter and darker circles indicate earlier and later years in that period respectively.

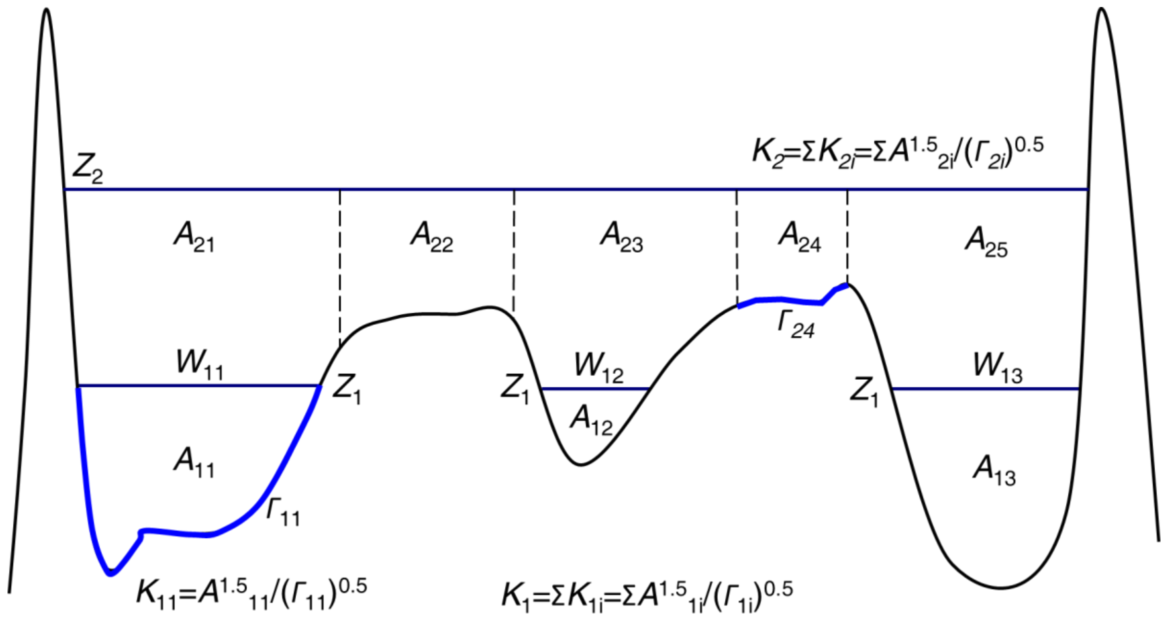

Fig. S7.

Sketch for the computation of geometric conveyance factor in the compound, multi-threaded channel-floodplain complex. Two flood stages Z_1 and Z_2 are used here as examples. When the flow stage is low (Z_1), the multi-threaded feature emerges and the geometric conveyance factor K_1 is computed as the sum of each subchannel; when the flow stage is high (Z_2), all channel and adjacent flood plains are added.

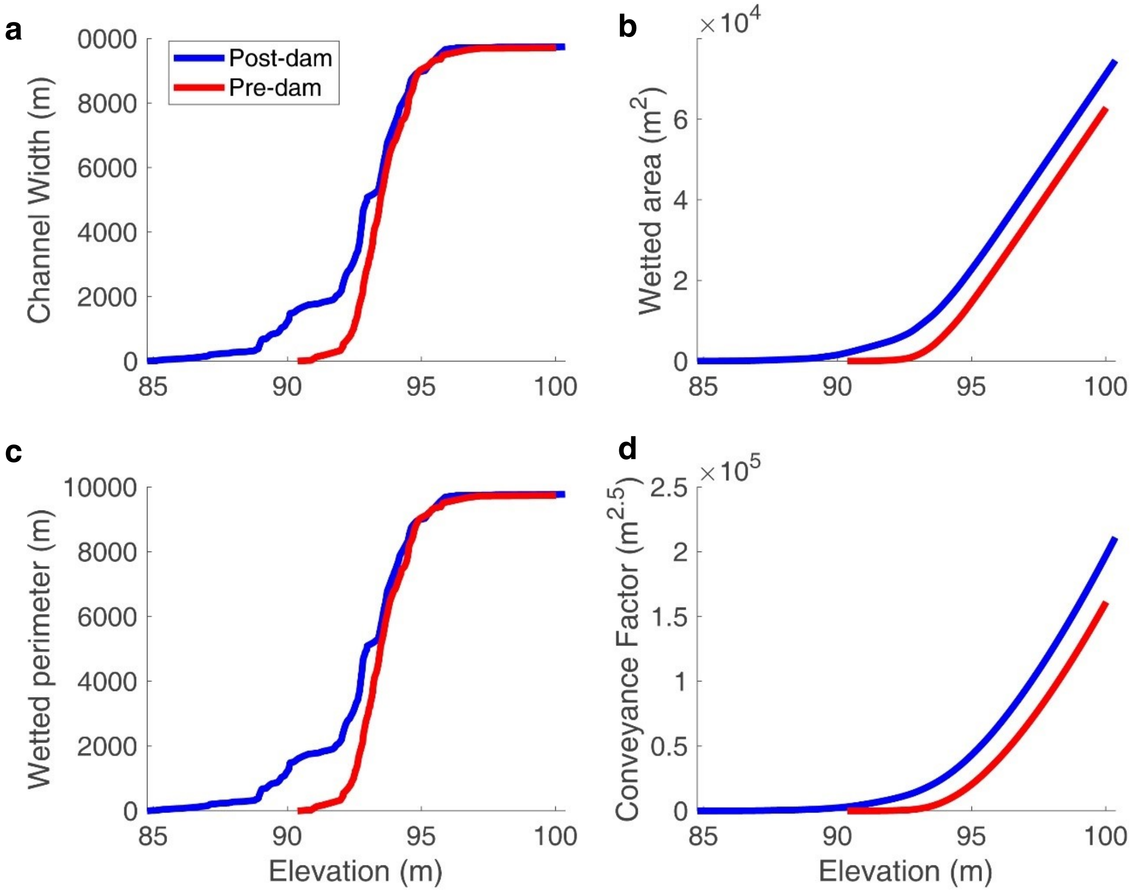

Fig. S8.

Examples of computations of channel geometry and geometric conveyance factor with respect to flow stage. The pre-dam case is computed from the cross-sectional data of 1981 and the post-dam case is computed from data of 2015 (Fig. 5a). Obvious post-damming channel widening and incision can be observed in (**a**) and, as a result, the post-dam wetted area (**b**), perimeter (**c**) and geometric conveyance factor (**d**) all increase significantly.

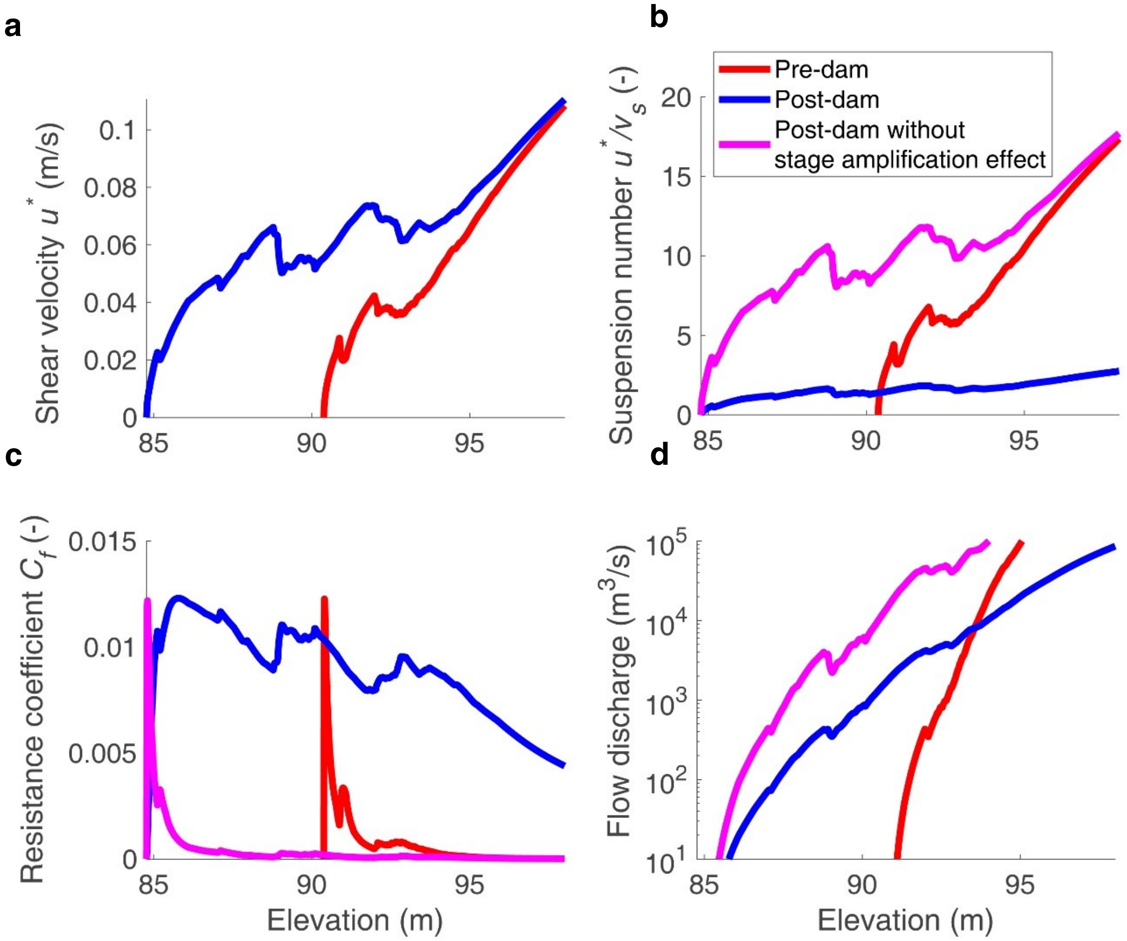

Fig. S9.

Examples of computations of hydraulic factors with respect to flow stage. The pre-dam case is computed based on the cross-sectional data of 1981 and the post-dam case is based on the cross-sectional data of 2015 (Fig. 5a). The post-damming incised and deepened channel leads to greater shear stress at the same flow stage (**a**); however, the change in grain size (*c.* from 0.09 to 0.3 mm), resulting in greater settling velocity, quickly reduces post-dam suspension number (**b**) and the lower suspension number post-damming sets the greater resistance coefficient (**c**). The overall conveyance of the post-dam channel is only increased in the small flood but is reduced for moderate and large floods after the stage amplification effect, caused by resistance enhancement, is considered (**d**).

Table S1. Summary of databases used in this study.

Databases	Description	Collection Location(s)	Collection Period	Data used/presented in Figure(s)	Collection Technique(s)
Cross-sectional average bed median grain size	Annual average median grain size with available data	Huayuankou, Jiayetan, Gaosun, Sunkou, Aishan, Luokou, Lijin (Fig. 1a)	1965-2015	Fig. 1c&d	See Text S1
Thalweg bed surface median grain size	Core collection with refined grids (~10 km-20 km spaced) focused on the river reach from Huayuankou to Gaocun	Wuhui, Yuanyang, Kaifeng, Dongbatou, Jiaoyuan, Changxing, Dongming (Fig. 1 a&b)	2016	Fig. 1d	See Text S2
Global bedform database	Bedform height, hydraulics information and grain size are documented	Global database including both field and laboratory data	-	Fig. 2 Fig. S2 Fig. 4b	See Refs. (39-40) and Text S5
Bed topography at the lower Yellow River	Bedform topography data collected with Multibeam Echo Sounder and Parametric Echo Sounder	Huanyuankou and Lijin at the lower Yellow River (Fig. 1a)	2015−2018	Fig. 3, Fig. S3	See Text S4 in and Table S3
Equilibrium hydraulic and sediment transport database	The equilibrium database includes water depth, channel width, discharge, water surface slope, bed grain size and sediment concentration. The data were carefully selected such that the hydraulic and sediment data were reproducible	Huayuankou and Lijin (Fig. 1a)	1980-1990 2001-2002	Fig. 4 a&b and Fig. S6	See Text S3
Routine hydrologic database	The database includes water depth, channel width, discharge, water stage and surface slope. The cross-section shape is measured twice a year	Huayuankou and Lijin (Fig. 1a)	1981-1990 2006-2015	Fig. 4c, Fig. 5 and Fig. S4	See Text S1
Global bankfull database	Bankfull geometry, hydraulics and bed grain size.	Global database	-	Fig. 6	See Refs. (55-56)
Cross-section profiles	Cross-section shapes at Huayuankou	Huayuankou (Fig. 1a)	1980-1990, 2006-2015	Figs. 5, S8 and S9	See Text S1

Table S2. Ranges of physical factors in the global bedform database.

H (m)	S (10^{-6})	D_{50} (mm)	U (m/s)	H_d (m)	u^*/v_s	H_d / H	C_f (10^{-3})
0.058-60.36	4-9400	0.13-36	0.31-2.66	0.006-7.5	0.116-8.231	0.0435-0.766	0.34-19.5

Table S3. Descriptions of bed topography surveys and the corresponding hydraulic, grain size and bedform geometry data.

Year	Location of Bed Topography Survey	Survey Instrument(s)	Mean H_d/H (%)	Thalweg D_{50} (mm)	Q (m^3/s)	S	H (m)	u^* (m/s)	v_s (m/s)	u^*/v_s
2015	Lijin	Multibeam	5.51 ± 3.26	0.09	2,180	1.3×10^{-4}	3.08	0.063	0.0062	10.10
2016	Lijin	Multibeam	11.68 ± 6.95	0.09	403	6.4×10^{-5}	2.03	0.036	0.0062	5.76
2016	Huayuankou	Multibeam/PES	26.88 ± 8.38	0.262	1,230	1.6×10^{-4}	3.28	0.072	0.0338	2.12
2017	Huayuankou	Multibeam	40.81 ± 12.72	0.27	607	1.4×10^{-4}	2.16	0.054	0.0351	1.55
2018	Huayuankou	Multibeam	14.27 ± 8.33	0.213	4,170	2.0×10^{-4}	5.3	0.102	0.0254	4.01

Table S4 Comparison of different predictive relations for bedform heights. Discrepancy Ratio (DR) is the ratio of the predicted value to the measured value, the mean of which indicates the systematic error of the method (unity denotes no systematic discrepancy); The Root-Mean-Square-Error (RMSE) of DR indicates the overall the uncertainty of the method (unity denotes no uncertainty).

Name	Range of Applicable	Mean Discrepancy Ratio	RMSE of Discrepancy Ratio	Within a factor of 1.25 (%)	Within a factor of 1.5 (%)	Within a factor of 1.75 (%)	Within a factor of 2.0 (%)	Within a factor of 3.5 (%)	Test against data from the LYR
Present	$0.1 < u^*/v_s < 10$	1.02	1.64	36.38	61.85	77.66	85.01	97.68	Fig. 2
Karim (1995)[29]	$0.15 < u^*/v_s < 3.65$	0.66	2.16	23.71	37.00	47.00	58.71	87.29	Not applicable*
Bradley & Venditti (2019)[27]	$0.27 < u^*/v_s < 1.65$	0.91	1.90	38.99	63.25	75.93	82.28	93.47	Not applicable*
Bradley & Venditti (2017)[39]	when water depth available	0.93	1.87	27.26	46.28	63.38	76.06	96.58	Fig. S5

*Not appliable: the relations predict negative values for the dune heights.

Table S5 Comparison of different predictive relations for resistance coefficients. Discrepancy Ratio (DR) is the ratio of predicted value to the measured value, the mean of which indicates the systematic error of the method (unity denotes no systematic discrepancy); The Root-Mean-Square-Error (RMSE) of DR indicates the overall the uncertainty of the method (unity denotes no uncertainty).

Name of Method	Mean Discrepancy Ratio	RMSE Discrepancy Ratio	Within a factor of 1.25 (%)	Within a factor of 1.5 (%)	Within a factor of 1.75 (%)	Within a factor of 2.0 (%)	Within a factor of 3.5 (%)
Present	1.09	1.55	47.26	69.80	82.28	86.87	98.91
Engelund & Hansen (1967)[38]	1.09	1.77	33.26	56.85	73.03	80.67	95.06
Wright & Parker (2003)[75]	0.51	1.61	8.31	19.33	29.44	39.33	95.51
